# Supplementary material for: Chitinase genes from Metarhizium anisopliae for the control of whitefly in cotton
Source: R Soc Open Sci. 2019 Aug 28;6(8):190412. doi: 10.1098/rsos.190412 (PMC6731705; doi:10.1098/rsos.190412)
Supplement: Supplementary data 1 file [file rsos190412supp1.docx]

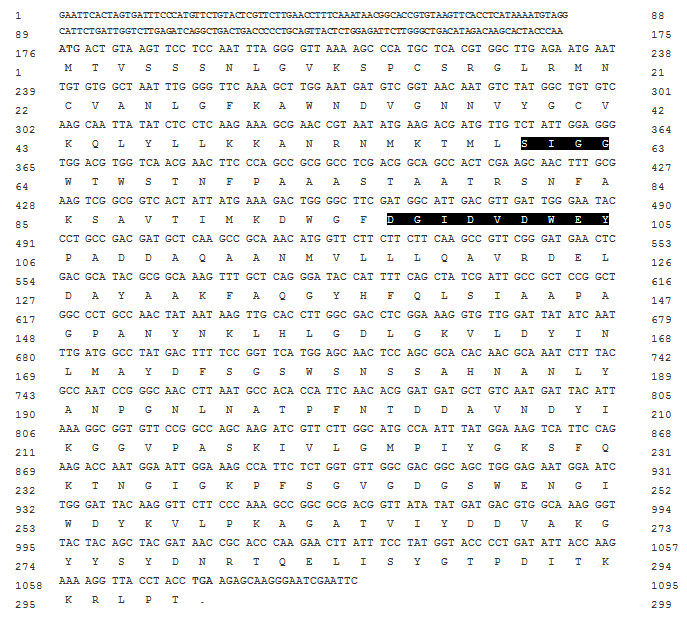


**Figure 1: Nucleotides along deduced amino acids indicated below codons of Met_*Chit1* gene of *M. anisopliae* isolate Tn-16. Substrate binding and catalytic domains are highlighted while start and stop codons are represented in bold**
