# Supplementary material for: Chitinase genes from Metarhizium anisopliae for the control of whitefly in cotton
Source: R Soc Open Sci. 2019 Aug 28;6(8):190412. doi: 10.1098/rsos.190412 (PMC6731705; doi:10.1098/rsos.190412)
Supplement: Supplementary data 2 file [file rsos190412supp2.docx]

**
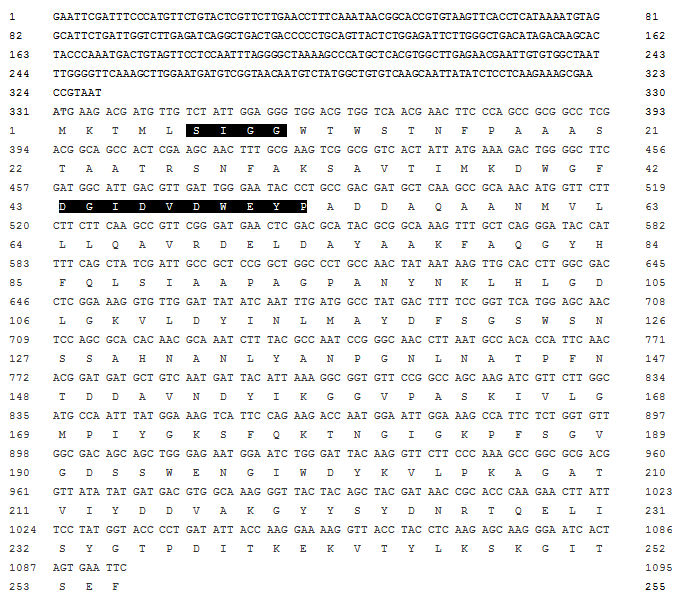
**

**Figure 2: Nucleotides along deduced amino acids indicated below codons of Met_*Chit1* gene of *M. anisopliae* isolate Tn-25. Substrate binding and catalytic domains are highlighted while start codon is represented in bold.**
